# Ciprofloxacin-Loaded Titanium Nanotubes Coated with Chitosan: A Promising Formulation with Sustained Release and Enhanced Antibacterial Properties

**DOI:** 10.3390/pharmaceutics14071359

**Published:** 2022-06-27

**Authors:** Soada Asadi, Bardia Mortezagholi, Alireza Hadizadeh, Vitaliy Borisov, Mohammad Javed Ansari, Hasan Shaker Majdi, Azizakhon Nishonova, Hossein Adelnia, Bahareh Farasati Far, Chaiyavat Chaiyasut

**Affiliations:** 1 Department of Anlytical Chemistry, Faculty of Chemistry, Urmia University, Urmia 5756151818, Iran; soada594@gmail.com; 2 Faculty of Dentistry, Islamic Azad University Tehran Branch, Tehran 1148963537, Iran; bardiamortezagholi@gmail.com; 3 School of Medicine, Tehran University of Medical Sciences, Tehran 1419733141, Iran; ali1375hadi@gmail.com; 4 Research Center for Advanced Technologies in Cardiovascular Medicine, Cardiovascular Diseases Research Center Institute, Tehran University of Medical Sciences, Tehran 1419733141, Iran; 5 Department of Propaedeutics of Dental Diseases, Sechenov First Moscow State Medical University, 119991 Moscow, Russia; vitaly.v.borisov@mail.ru; 6 Department of Pharmaceutics, College of Pharmacy, Prince Sattam Bin Abdulaziz University, Al-Kharj 11942, Saudi Arabia; javedpharma@gmail.com; 7 Department of Chemical Engineering and Petroleum Industries, Al-Mustaqbal University College, Babylon 51001, Iraq; hassanshaker1@gmail.com; 8 Department of Physiology and Pathology, Tashkent State Dental Institute, Tashkent 100047, Uzbekistan; b.abdullaev@editory.org; 9 Australian Institute for Bioengineering and Nanotechnology, The University of Queensland, Brisbane, QLD 4072, Australia; h.adelnia@uq.net.au; 10 Department of Chemistry, Iran University of Science and Technology, Tehran 1684613114, Iran; 11 Innovation Center for Holistic Health, Nutraceuticals, and Cosmeceuticals, Faculty of Pharmacy, Chiang Mai University, Chiang Mai 50200, Thailand

**Keywords:** titanium dioxide nanotube, anodic oxidation, chitosan, antibacterial, drug delivery

## Abstract

Due to their high entrapment efficiency, anodized titanium nanotubes (TiO_2_-NTs) are considered effective reservoirs for loading/releasing strong antibiotics whose systemic administration is associated with diverse and severe side-effects. In this study, TiO_2_-NTs were synthesized by anodic oxidation of titanium foils, and the effects of electrolyte percentage and viscosity on their dimensions were evaluated. It was found that as the water content increased from 15 to 30%, the wall thickness, length, and inner diameter of the NTs increase from 5.9 to 15.8 nm, 1.56 to 3.21 µm, and 59 to 84 nm, respectively. Ciprofloxacin, a highly potent antibiotic, was loaded into TiO_2_-NTs with a high encapsulation efficiency of 93%, followed by coating with different chitosan layers to achieve a sustained release profile. The prepared formulations were characterized by various techniques, such as scanning electron microscopy, differential scanning calorimetry, and contact measurement. In vitro release studies showed that the higher the chitosan layer count, the more sustained the release. Evaluation of antimicrobial activity of the formulation against two endodontic species from *Peptostreptococcus* and *Fusobacterium* revealed minimum inhibitory concentrations (MICs) of 1 µg/mL for the former and the latter. To summarize, this study demonstrated that TiO_2_-NTs are promising reservoirs for drug loading, and that the chitosan coating provides not only a sustained release profile, but also a synergistic antibacterial effect.

## 1. Introduction

For many years, biological implant materials have been a cause of discomfort in clinical therapy, as millions of implants are employed in orthopedics and dental surgeries, and cosmetology [1,2]. Implants may cause complications such as inflammation, bone loss, infection, and others. Infection is one of the most significant issues of implants which can culminate in osteomyelitis and implant failure [3]. Currently, bacterial infection is treated mainly through systemic administration of antibiotics, either orally or intravenously. Although widely practiced, this could still be insufficient due to the low efficacy of some antibiotics, besides their adverse effects, which are often difficult to resolve [4]. Additionally, a biofilm on the implant could prevent proper integration of the implant to the tissue, thereby resulting in its failure [5,6]. To overcome the limitations of conventional drugs, such as limited solubility (hydrophobic and water-insoluble), failure to selectively target the disease site, adverse effects, and undesirable pharmacodynamics, many studies have been conducted in recent years toward developing more effective drug delivery systems, highlighting the importance of a high level of biocompatibility, loading capacity, and unique structural properties [7,8].

Titanium (Ti) and its alloys, which are currently used in clinical surgeries, are promising candidates for this purpose, as they are extremely durable, nonreactive to human tissues (e.g., dental and bone), and incompatible with living tissue [9,10,11,12]. Despite these, they can neither form strong bonds with bone during the early stages of osseointegration, nor carry/deliver bioactive agents and provide sustained release. Numerous studies have thus been conducted to address such issues through surface modification techniques, such as anodic oxidation [13,14,15]. The anodically oxidized titanium nanotubes (TiO_2_-NTs) are highly attractive for drug delivery systems due to their biocompatibility, high specific surface area, and excellent physicochemical properties [16]. Compared to solid drug nanocarriers, hollow TiO_2_-NTs offer a much higher drug loading volume and slower drug release kinetics as a result of efficient drug entrapment [14]. In addition, surface coating of titanium nanostructure-based implants with optimum dimensions can significantly improve osseointegration by incrementing specific surface area, modification of surface topography, and establishing strong bonds with bone tissue. TiO_2_-NTs with the optimum length in cell adhesion were shown to drive migration of both osteoblast and mesenchymal stem cells, reinforcing the contacts between cells and implant surfaces [17,18].

In previous studies, silver nanoparticles were deposited on NT arrays to increase the antibacterial properties [19]. In another study, the NTs were loaded with penicillin-based antibiotics using a co-precipitation technique, which increased antibacterial activities and thus treatment efficacy [20]. However, techniques that include antibacterial agents directly on the surfaces of implants often fail due to rapid release of the drug and a short duration of the antibacterial effect [21]. To tune the drug release, gentamicin-sulfate-loaded TiO_2_-NTs were coated with a thin chitosan layer. These prepared NTs exhibited antibacterial activity and a high degree of controlled release [22]. Another study also developed TiO_2_-NT loaded with cefuroxime coated with chitosan, which exhibited strong antibacterial activity and sustained release over 7 days. The thickness of the chitosan coating played an important role in the rate of cefuroxime release [23]. In another study, bacterial adhesion and proliferation were strongly inhibited both in vitro and in vivo by TiO_2_-NTs loaded with hyaluronic acid and gentamicin. At the same time, the osseointegration features of the nanostructured surface were well maintained without any negative impact [24].

Ciprofloxacin (CIP) is a highly potent quinolone derivative with a broad antibacterial spectrum that makes useful for applications from endodontic work [25] to bone infections [26], and is typically used as a last treatment option, especially for infections that are resistant to conventional antibiotics [27]. CIP, especially at high doses, can have substantial adverse effects, including tendon problems [28], nerve damage [29], severe mood or behavior changes [30], and hypoglycemia [31], which can potentially be mitigated by a controlled release profile from an efficient delivery system.

**Scheme 1:**
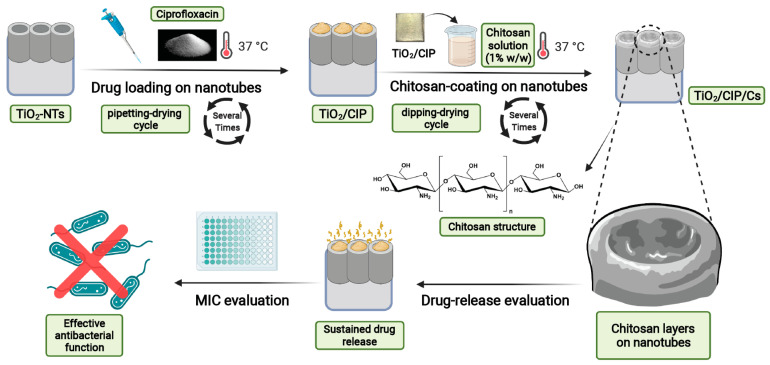
Schematic representation of the procedure for loading of CIP, coating with chitosan, sustained release, and antibacterial activity of the prepared formulation.

This study synthesized TiO_2_-NTs via anodic oxidation in light of the above discussion. Various parameters involved in synthesizing TiO_2_-NTs, such as electrolyte percentage and viscosity, were studied. The NTs served as effective reservoirs for CIP storage and controlled release. The NTs loaded with CIP were then coated with chitosan (Cs) to tune the release. Cs is well-known as a biocompatible and biodegradable polymer with an inherent antibacterial effect [32]. However, previous studies are not in agreement in terms of the effect of the number of Cs layers applied to TiO_2_-NTs on the release profile [33,34]. Thus, the effect of Cs thickness on the release was also investigated. The prepared formulation exhibited a synergistic antibacterial effect on Gram-positive and negative bacteria for which there is no report on the impact of CIP, to the best of our knowledge. Overall, as depicted in Scheme 1, this study presents a simple, cost-effective approach for developing biocompatible implants with synergistic antibacterial effects.

## 2. Materials and Methods

### 2.1. Materials and Methods

Titanium foils (with a thickness of 3 mm, a diameter of 15 mm, and 99% purity, Naothang Metal Co, Ltd., Hongkong, China) were purchased from AppliChem. Chitosan (190–310 kDa, deacetylation degree of 80–85%), potassium chloride, ammonium-fluoride, ethylene glycol, acetic acid, potassium hydrogen phosphate, disodium hydrogen phosphate, sodium chloride, and sodium hydroxide were purchased from Sigma-Aldrich. CIP with the formula C_17_H_18_FN_3_O_3_ HCl H_2_O was obtained from Temad Co., Ltd. (Karaj, Iran). 

### 2.2. Synthesis of TiO_2_-NTs

To synthesize TiO_2_-NTs by the anodizing method, titanium sheets with dimensions of 15 × 15 × 3 mm were cut, followed by sanding the foil surface, and subsequent ultrasonic distillation in a solution containing ethanol, acetone, and distilled water. After air-drying of the foils, they served as the anode. At the same time, platinum was used as the cathode, which was attached to a fixed potential supply device (Programmable Switching D.C. Power Supply Model PSP-603) and then inserted into the electrolyte. Different amounts of water as an electrolyte were used to study its effects on the nanotube wall’s thickness, crater radius, and length. Several electrolytes containing ammonium fluoride, water, and ethylene glycol were made. Electrodes were placed in a 2-electrode system with a distance of 20 mm between the electrodes and a temperature of 25 °C. CaCl_2_ (0.1% *w*/*w*) was added to the electrolyte for the improvement of the ionic strength [35,36]. The percentage of ammonium fluoride in all samples was 0.35% *w*/*w*, and the percentage of water varied. The procedure was performed at a constant potential voltage of 50 V for 2 h. Following anodizing, the synthesized nanotubes were washed with water, then immersed overnight in distilled water and pure ethanol in sequence. Two-hour annealing was carried out at 350 °C (10 °C/min) after air drying, followed by progressive cooling.

### 2.3. Characterization of TiO_2_/CIP/Cs

The possible interactions between the components within the polymeric system were studied by Fourier transform infrared (FT-IR) spectroscopy via a KBr pellet procedure. FT-IR spectra of Cs, TiO_2_-NTs, CIP, and TiO_2_/CIP/Cs were recorded using a PerkinElmer Spectrum Version 10.03.06. The nanocomposite’s surface morphologies were investigated using the KYKY-EM3200 field-emission scanning electron microscope (FE-SEM) at an accelerated voltage of 25 KV. As part of our surface characteristics, we used a camera (Canon EOS Rebel XS, Tokyo, Japan) and a macro lens (105 mm F2.8 EX DG OS, Sigma, St. Louis, MI, USA) to collect ten sessile drop measurements from each group. Contact angle (CA) was measured using the 10 μL dH_2_O droplets’ profiles created using the software Digimizer, which produced the changed surfaces immediately after stabilization. Energy-dispersive X-ray spectroscopy (EDX) was performed to chemically analyze Cs coated TiO_2_-NTs’ surfaces (JEOL JSM-5910LV, Tokyo, Japan) using a scanning electron microscope.

### 2.4. The Effect of Electrolyte Water Percentage on the Morphology of TiO_2_-NTs 

To investigate the effect of water, several electrolytes containing ammonium fluoride, water, and ethylene glycol were made. Ammonium fluoride content was 0.35% in all samples. The percentage of water changed (15, 20, 25, 30% *w*/*w*). 

### 2.5. Drug Encapsulation and Polymer Coating on the TiO_2_-NTs

Pipetting and vacuum drying were used to insert the CIP into the nanotubes. The CIP (40 mg/mL) solution was prepared by dissolving CIP in deionized water. Noteworthy is that CIP salts are partially soluble in water [37]. For drug encapsulation into the TiO_2_-NTs, 100 μL of CIP solution was pipetted onto the TiO_2_-NTs’ surface. Following pipetting, samples were dried in a vacuum oven set at 35 °C for 4 h. The deposition and evaporation steps were repeated 20 times. After final drying, the sample surfaces were washed with 1 mL PBS to eliminate weakly bound CIP. Subsequently, Cs solution (1% *w*/*v*) was generated by dissolving Cs in a 1% (*v*/*v*) acetic acid solution. A dipping procedure was used to cover the samples with a surface coating to prevent drug burst release. The drug-loaded samples were dipped in Cs solution and dried in a vacuum oven at 40 °C. The dipping process was completed quickly (3 min each time) to prevent CIP diffusion into the Cs solution. This process was repeated up to 10 times to achieve the desired thickness for sustained release of CIP. Equation (1) was used to determine the encapsulation efficiency:

(1) $$\mathrm{EncapsulationEfficiency}\left(\%\right)=\frac{\mathrm{Encapsulated}\;\mathrm{CIP}\;\mathrm{Concentration}}{\mathrm{Initial}\;\mathrm{CIP}\;\mathrm{concentration}}\left(\mathrm{mg}\right)\times 100$$

### 2.6. Investigation of the Effect of Chitosan Layer Thickness on Release Behavior

To discover an optimal layer thickness for the extended release of drugs, we tested samples with varied coating thicknesses (and dipping cycles) to identify the optimal sample in terms of sustained release [23]. Chitosan films of various thicknesses were applied to anodized TiO_2-_NTs to reduce the effect of chitosan thickness on the release of drugs (T15%). Chitosan solution (1%) was applied to the Ti sheets for 30 s and air-dried. We will use the terms “TiO_2_/C0”, “TiO_2_/C3”, “TiO_2_/C5”, “TiO_2_/C8”, and “TiO_2_/C10”, respectively, to describe the non-coated, three-layered, five-layered, eight-layered, and ten-layered samples [33]. Ellipsometer measurements were used to evaluate the thicknesses of the chitosan films on the samples (Si-wafer as the control sample).

### 2.7. Investigation of Drug Release from Polymer-Coated TiO_2_-NTs

The CIP release from TiO_2_-NTs and Cs-coated TiO_2_-NTs was investigated by placing the samples in a PBS solution (pH 7.4) at 37 °C. The absorbance of the solution was obtained by a UV–Vis spectrophotometer at 270 nm. (concentrations in mg). Equation (2) represents the cumulative release calculation:

(2) $$Q=\frac{C_{n}\times V_{0}+V_{i}\sum_{i=1}^{n-1}Ci}{m}\times 100\%$$

where *Q* is the cumulative drug release (%), *C_n_* is the mass concentration of drug released, *V*_0_ is the total volume of PBS, *V_i_* is the volume removed per unit of time, *C_i_* is the concentration of drug in the volume removed per unit of time, and m is total drug loaded.

### 2.8. Kinetic of Release

#### 2.8.1. Zero-Order Model 

Equation (3) can be used to depict the dissolving of drugs from nondisaggregating dose forms that release the drug slowly:

(3) $$m_{t}={\;m}_{b}+{\;k}_{0}t$$

where m_t_ is the CIP amount released at time t, m_b_ is the CIP amount in solution before release (usually t = 0), and k_0_ is the zero-order release rate constant.

The cumulative CIP release was plotted against time to investigate the release kinetics. 

#### 2.8.2. First-Order Model

The first-order model, as described by Equation (4), has also been utilized to explain the absorption and elimination of specific drugs.

(4) $$\log C\;=\log C_{0}k_{t}/2.303$$

where K is the first-order rate constant represented in 1/s units, C_0_ is the initial CIP concentration, and t is the time. The acquired data were plotted as cumulative log percentage of remaining drug vs. time, which resulted in a straight line with a slope of K/2.303.

#### 2.8.3. Higuchi Model

The Higuchi model defines drug release from the scaffold as the square root of a time-dependent process governed by Fick’s diffusion rule (Equation (5)). It is denoted as:

(5) $$m_{t}={\;\mathrm{kt}}^{0.5}$$

where k is a constant, m_t_ is the weight of CIP released at time t, and t is the time. The data obtained from the drug release are represented as the cumulative percentage of drug released vs. the square root of time. Correlation coefficients with higher values indicate a diffusion control mechanism for drug release.

#### 2.8.4. Korsmeyer–Peppas Kinetics

In Korsmeyer–Peppas’ model, the process of drug release from the matrix is represented by a value (n) which is a diffusional exponent (Equation (6)). When n = 0.5, the drug diffusion follows a quasi-Fickian mechanism. When n > 0.5, the drug’s diffusion mechanism is anomalous or non-Fickian, and when n = 1, the drug’s release kinetics are non-Fickian, zero-order, or case-II.

(6) $$\log\left(\frac{m_{t}}{m_{\infty }}\right)=\;\mathrm{logk}\;+\;\mathrm{nlogt}$$

### 2.9. Antibacterial Experiment 

#### 2.9.1. Bacterial Strains 

For antibiotic susceptibility assays, sterile swabs were used to pick bacteria—from abscess infections, mostly. Eight bacterial strains were isolated from 15 abscess-infected patients (seven-man and eight-woman). Samples were first diluted and then cultured in Müller Hilton blood agar and thioglycollate under anaerobic conditions (CO_2_ incubator) for 48 h on Brucella blood agar plates; subsequently, numerous colonies were inoculated in 10 mL of Brucella broth suspension and incubated anaerobically at 37 °C for 6 h [38]. Gram staining tests and biochemical tests identified grown specimens. Among anaerobic bacteria, *Peptostreptococcus* and *Fusobacterium* were selected for the antibacterial test, as they are two of the most important bacteria in dental abscess infections [39].

#### 2.9.2. Determination of Minimum Inhibitory Concentrations (MICs)

As the most commonly observed bacteria in dental infections, *Peptostreptococcus* and *Fusobacterium* were used to determine the antibacterial effects of the collected samples: MICs of TiO_2_-NTs, CIP, TiO_2_/CIP, and TiO_2_/CIP/Cs against *Peptostreptococcus* and *Fusobacterium* strains. The broth microdilution method was used to determine the isolates, interpreted according to CLSI (Clinical and Laboratory Standards Institute (Wayne, PA, USA) standards. Briefly, Mueller Hinton broth (Merck Co., Ltd., Hamburg, Germany) containing serial dilutions of CIP, TiO_2_-NTs, TiO_2_/CIP, and TiO_2_/CIP/Cs were made in the range of 0.062–64 μg/mL.

The bacterial densities of the suspensions were adjusted to be equal using sterile Brucella broth using the 0.5 McFarland standard, and 1 L was put in five separate locations onto prepared 1.5% agar and then added to 96-well plates. Selected bacterium suspensions equivalent to a 0.5 McFarland standard were placed into plates and cultured anaerobically for two days at 37 °C. In the case of broth microdilution, the MIC was defined as the lowest dilution that resulted in no growth. The MIC was determined as the lowest concentration of TiO_2_ (μg/mL) that prevented observable microorganism growth. After an incubation time, the MIC value was visually determined. Colony-forming units per milliliter (CFU mL^−1^) were obtained after 24 h of incubation at 37 °C on brain–heart infusion agar plates [40].

#### 2.9.3. Determination of Minimum Bactericidal Concentration (MBCs) 

Using the MIC results from all samples, 100 μL aliquots at concentrations from 64 to 0.06 μg/mL were tested. The diluted agents were mixed with a standardized suspension (100 μL) of each strain, seeded on Muller Hinton Broth agar plates, and cultured for 48 h at 37 °C using a CO_2_ incubator. The presence or absence of bacteria on pre-and post-incubated agar plates was determined. It is referred to as an MBC endpoint when an active ingredient kills 99.9% of the bacteria at the lowest possible concentration.

#### 2.9.4. Assessment of Biofilm Formation

The crystal violet staining technique was used to analyze the biofilm load of bacterial strains treated with all samples (TiO_2_-NTs, CIP, TiO_2_/CIP, and TiO_2_/CIP/Cs). Pre-experiment adjustments were made to bacterial strains so that they could be used in the experiment. Each well of a 96-well plate was pre-incubated for 24 h at 37 °C with 1% glucose in a CO_2_ incubator to allow the biofilm’s formation. Incubation of samples (10 mg/mL) in the preexisting biofilms at 37 °C for an additional 24 h was followed by several washings with DI water. Then, it was stained with 0.1% crystal violet solution for 30 min and gently washed with sterile, DI water. For the next 15 min, the plate was incubated at 37 °C with distilled water and washed once more. For 15 min, 95% ethanol was used to elute crystal violet that had been attached to the biofilm. The negative control was 95% ethanol (blank). According to the results, the reading at 570 nm was used. Blank readings (570 nm) were subtracted from the sample values to correct for background absorbance.

### 2.10. Hemolysis Assay 

A healthy volunteer’s blood was centrifuged at 2000 rpm for 5 min to collect red blood cells (RBC). The red blood cells were added to a 2% physiological saline (37 °C) mixture and washed three times to generate the clear supernatant. The solution was then centrifuged for 6 min at 3000 rpm. Various concentrations (100, 75, and 50 μg/mL) of TiO_2_/CIP/Cs nanoformulation in PBS (0.7 mL) were combined with dilute RBC solution (0.3 mL) and stored at room temperature for 2 h without shaking. PBS was the negative control. After 2 h, absorbance at 540 nm was measured using UV–visible spectroscopy and centrifugation to assess the ratio of hemolysis in the supernatant of each sample. The following equation (Equation (7)) was used to estimate the percentage of hemolysis in each sample:

(7) Percent Hemolysis(%):(sample absorbance−negative control absorbance/positive control absorbance−negative control absorbance)×10

### 2.11. Statistical Analysis 

The means and standard deviations (SD) of experimental results are reported. The one-way ANOVA test with the post hoc Tukey method was utilized to compare conditions. A value of *p* < 0.005 was considered statistically significant by SPSS (V. 17). 

## 3. Results

### 3.1. Characterization of TiO_2_/CIP/Cs 

**Figure 1:**
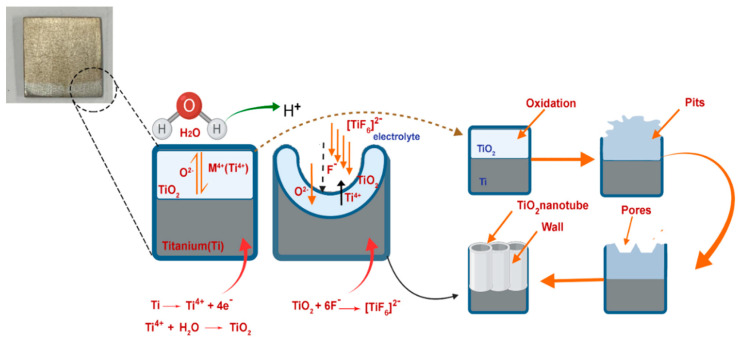
Mechanism of anodic oxidation.

**Figure 2:**
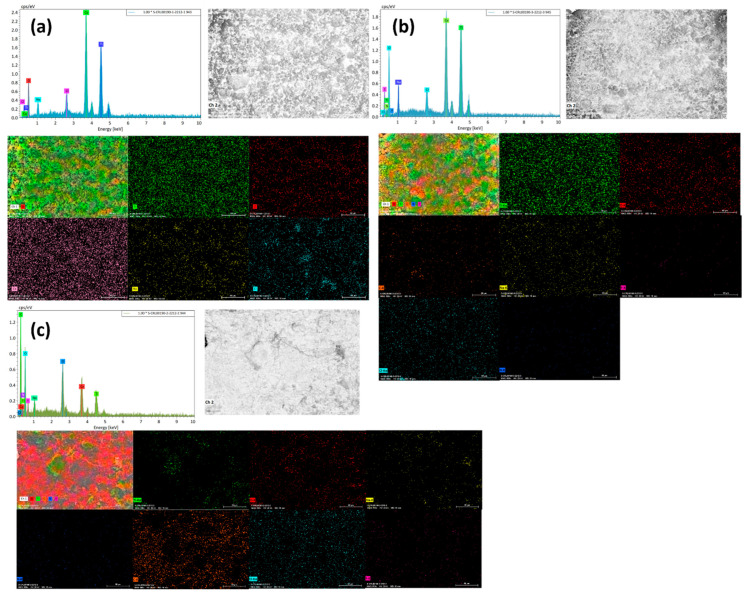
EDX spectra of (**a**) TiO_2_-NTs, (**b**) TiO_2_/CIP, and (**c**) TiO_2_/CIP/Cs.

The electrical anodization method was adopted to develop the TiO_2_-NTs (Figure 1). As shown in Table 1, the EDX results suggest successful synthesis of TiO_2_-NTs with 64.29% O and 14.52% Ti. The peaks of calcium are related to electrolyte enhancer, which remained in the structure of TiO_2_-NTs. To validate that CIP was loaded into the NTs, the chemical compositions of CIP-loaded TiO_2_-NTs samples were also determined by EDX, as illustrated in Figure 2. As presented in Table 1, Ti, O, C, F, Ca, Na, and Cl elements were visible on the cross-sectional surfaces of both samples. Impurities led to the appearance of new peaks, such as Na, Si, and Ca signal s, in the sample. The simultaneous appearance of strong Ti and O signals corroborated the successful synthesis of TiO_2_-NTs. The EDX spectrum also revealed the presence of other elements, such as Cl, which originated from the hydrochloride salt of CIP employed in the investigation [41]. The presence of F in the EDX spectrum was also attributed to the successful entrapment of CIP in the nanotubes. By Cs coating in the TiO_2_/CIP/Cs sample, in addition to the presence of previous atoms, the percentage of N increased due to NH_2_ groups in Cs, which suggests successful coating of Cs. 

**Figure 3:**
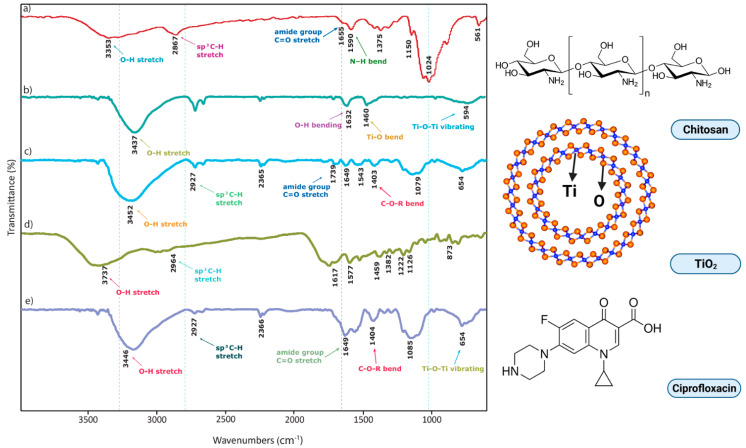
FTIR spectra of (**a**) chitosan, (**b**) TiO_2_-NTs, (**c**) CIP, (**d**) TiO_2_/CIP, and (**e**) TiO_2_/CIP/Cs.

Additionally, FTIR was utilized to analyze the chemical structures of the samples (Figure 3). The FTIR spectrum of the CIP-loaded TiO_2_-NTs showed successful incorporation of CIP, which could exist both around and inside the NTs. Cs has a significant band at 3353 cm^−1^ due to the OH and the symmetrical stretching of N–H bonds. The 2867 cm^−1^ peak is related to CH_2_ stretching [42]. The peak at 1024 cm^−1^ is due to the vibrations of C–O in Cs [43], whereas that centered at 1375 cm^−1^ is ascribed to asymmetrical C–H bending of the CH_2_ groups [44]. The peaks at 1655 and 1590 cm^−1^ are due to –C=O stretching (amide I) and NH stretching (amide II) [45].

The FTIR spectrum of TiO_2_-NTs showed a broad absorption band in the range of 400–600 cm^−1^ (centered at 594 cm^−1^) related to Ti–O–Ti stretching vibrations, suggesting successful synthesis of TiO_2_-NTs [46]. The band observed around 3437 cm^−1^ corresponds to the OH groups on the surfaces of TiO_2_-NTs samples, arising from the electrolyte molecules in the anodizing process. At 1632 cm^−1^, the adsorbed H_2_O bending mode and the Ti–OH mode are visible [41]. At 1460 cm^−1^, a band was formed by Ti–O modes. The bands at 3452 and 1649 cm^−1^ are due to hydroxyl groups of the absorbed water on the TiO_2_-NTss surfaces [47].

The absorption peaks at 1649 and 1543 cm^−1^ are assigned to the NHCOCH_3_ (amide I) and NH_2_ groups (amide II), respectively, confirming the presence of Cs on the surfaces of TiO_2_-NTs. In the FTIR spectrum of CIP, the peaks at 3737 and 1617 cm^−1^ are related to OH stretching and quinolone structure, respectively [47]. The peaks between 1382 and 1459 cm^−1^ belong to C–O; and the peaks at 1382 to 1222 cm^−1^ resulted from the OH group of carboxylic acid. The peaks related to the C-F group are at 1126 and 873 cm^−1^. The peak at around 3446 cm^−1^ corresponds to the OH groups on the surfaces of CIP-loaded TiO_2_-NT samples, presented to electrolytes during the anodized production process, and the OH group of Cs.

The FTIR spectrum of TiO_2_/CIP/Cs showed peaks 1649 and 654 cm^−1^, which are related to TiO_2_-NTs. The shift in the positions of these peaks was due to the bonding of TiO_2_-NTs to Cs. The peaks at 2366 and 1404 cm^−1^ in the spectrum of the composite further confirmed the presence of Cs. The shift in these peaks could have been due to the bonding between Cs and TiO_2_-NTs. The peak at 1085 cm^−1^ also indicates that CIP was successfully incorporated into the TiO_2_-NTs. Likewise, the shifts in the position of the characteristic peaks of CIP could be attributed to the interaction of CIP with Cs and TiO_2_-NTs.

### 3.2. DSC Analysis

**Figure 4:**
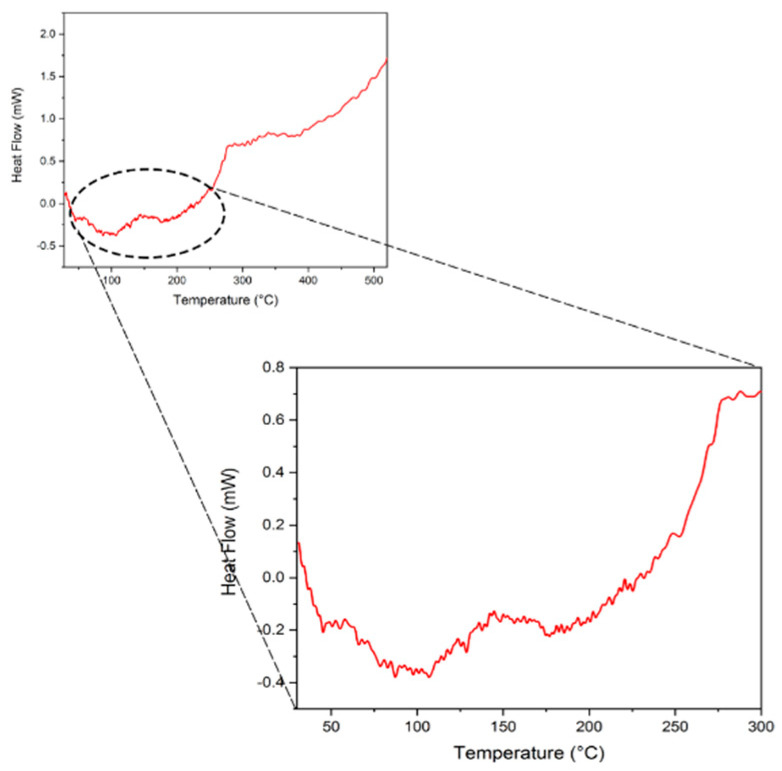
DSC curves of TiO_2_/Cs samples at a heating rate of 10 °C/min.

As presented in Figure 4, the DSC thermogram of Cs-coated TiO_2_-NTs exhibited a broad endothermic peak centered at around 100 °C, and two broad exothermic peaks at around 155 and 267 °C. The endothermic peak, referred to as the dehydration temperature, is the temperature range at which the absorbed water is lost through evaporation. Cs, as a hydrophilic polysaccharide with a disordered structure in its solid state (i.e., amorphous), has a high affinity for water, making it quickly hydrated upon exposure to ambient conditions and humidity. The presence of this peak verified the presence of the hydrophilic Cs on the TiO_2_-NTs. The exothermic peaks attributed to Cs heat breakdown (monomer dehydration, glycoside bond cleavage, and disintegration of acetyl and deacetylated units) [48] further suggest that the TiO_2_-NTs were coated successfully with Cs. 

### 3.3. Contact Angle

**Figure 5:**
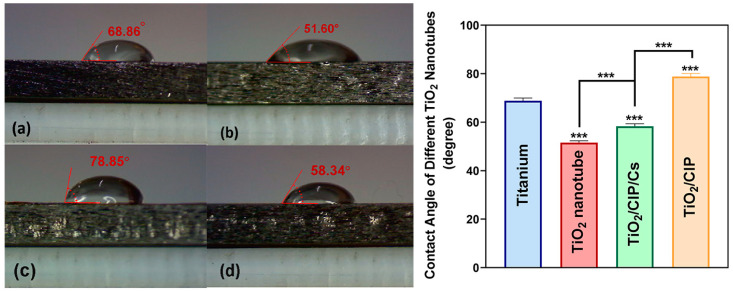
The wettability of Titanium (**a**), TiO_2_-NTs (**b**), TiO_2_/CIP (**c**), and TiO_2_/CIP/Cs (one layer) (**d**) prepared by anodic oxidation method. The contact angles for each group (mean ± SD, n = 6) (*** *p* < 0.001, ** *p* < 0.01, * *p* < 0.05).

Figure 5 shows the sketch maps used to determine the contact angle. On the anodized sample with nanotube arrays and Ti-substrate, the average contact angles of water are 51.60 and 68.86 degrees, respectively. Such a considerable decrease in contact angle after the reaction could suggest the nanotube array structure and oxidation of Ti collectively contribute to improving the wettability. Once the contact angle exceeds 20, the surface is regarded as hydrophobic [49]. Contact angle measurements (Figure 5) showed a statistically significant increase from TiO_2_-NTs, TiO_2_/CIP and TiO_2_ /CIP/Cs to Titanium (*p* < 0.001). There was a significant increase at TiO_2_/CIP/Cs compared to TiO_2_/CIP (*p* < 0.001). As noted, incorporation of CIP significantly increased the contact angle, while following the Cs coating, the contact angle decreased almost to that of neat TiO_2_-NTs. The former and the latter could be attributed to hydrophobicity and hydrophilicity of CIP and Cs, respectively.

### 3.4. Effect of Water Percentage of Electrolyte on Morphology

**Figure 6:**
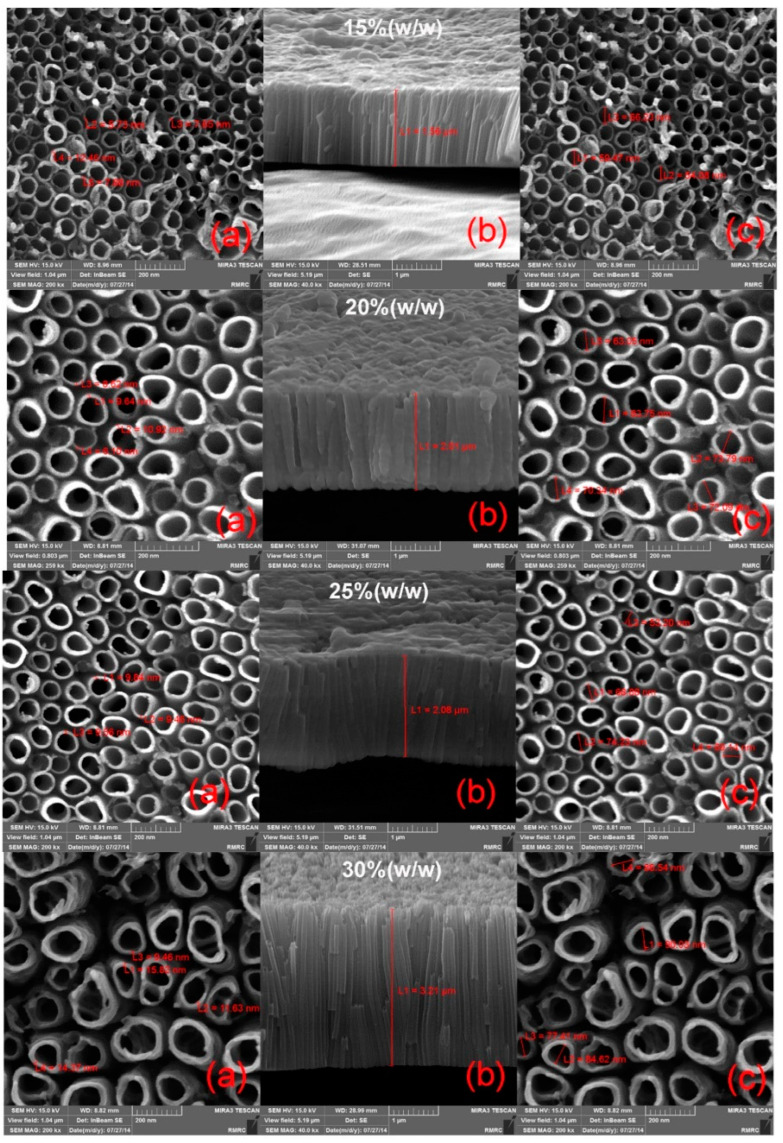
FESEM images of TiO_2_-NTs: wall thickness (**a**), cross-section image (**b**), and inner diameter (**c**). The were prepared via anodizing method using an electrolyte solution containing 15 to 30% of water, 0.35% *w*/*w* ammonium fluoride, calcium chloride, and ethylene glycol for 2 h at 50 V at room temperature.

Figure 6 illustrates the morphology of TiO_2_-NTs samples generated with varying percentages of water. The cross-sectional and top surface views of TiO_2_-NTs samples revealed that anodization produced highly ordered and vertically aligned TiO_2_-NTs arrays, having a unique pore structure with an average diameter in the range of 59–84 nm, wall thickness of 5.9–15.8 nm, and length of 1.56–3.21 µm (Table 2). As seen, increasing the water content increased the wall thickness, inner diameter, and length of the nanotubes.

In general, the viscosity of the electrolyte strongly affects the pore structure and size, as it leads the diffusion of the species present in the reaction to the surfaces of TiO_2_-NTs. The rate of nanotube formation in glycerol-based solvents (at 25 °C, ƞ = 945 cP) is much lower than that in ethylene glycol-based solvents (at 25 °C, 16 ƞ = 16 cP) due to the higher viscosity of former (approximately 60 times higher). As shown in Table 2, the addition of water to ethylene glycol increased the wall thickness, inner diameter, and length of the nanotubes.

### 3.5. Drug Release from TiO_2_-NTs

As mentioned before, Equation 1 was used to determine the entrapment efficiency rate of CIP hydrochloride. In different samples of TiO_2_-NTs, 83.19–92.95% of the drug was encapsulated in the TiO_2_-NTs (Table 3).

**Figure 7:**
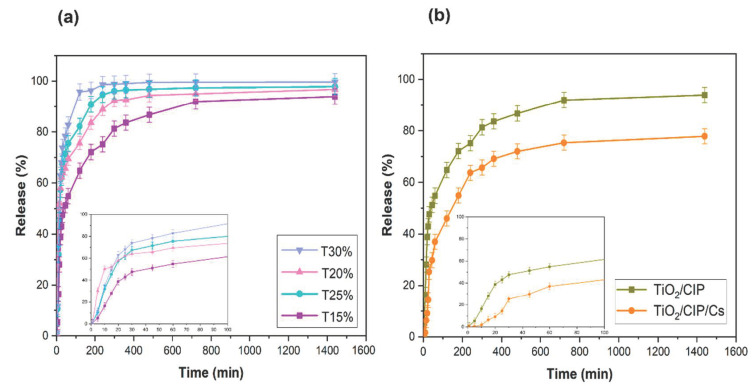
Release profile of (**a**) CIP loaded on the TiO_2_-NTs prepared at 50 V in an electrolyte containing 15, 20, 25, or 30% water; 0.35% ammonium; and ethylene glycol for 2 h. (**b**) Optimum TiO_2_/CIP selected and evaluated for release profile: bare and Cs-coated.

To evaluate the drug-releasing mechanism and the effect of polymer coating on the kinetics of release, CIP was released from TiO_2_-NTs in a PBS solution at pH 7.4 and 37 °C. Figure 7 represents the findings of CIP release experiments using Cs-coated TiO_2_-NTs. As seen, after 24 h in the TiO_2_-NTs release process, 99.21% of the drug was released. Drug release rate increased in the first few minutes and then declined over time: roughly 50% of the drug was released in the first 15 min, and the remaining drug content, or 99.21%, was released over approximately 24 h. This is related to the time required for the drug molecules to diffuse from the deeper areas of the NTs to the surrounding environment. Clearly, as the length of the NTs increased, the rate of drug release reduced.

As seen, CIP was instantly released from the neat TiO_2_-NTs sample (i.e., without Cs coating), but with the Cs, the release profile showed 37.95% release during the first 1 h, followed by slow release. From the major changes, it is obvious that that Cs coating markedly lowered the drug release rate. The burst release of 91.78% of the total amount loaded in 5 h in the TiO_2_-NTs profile suggested that the coating generated by five dipping times was sufficient to control the release, as it decreased to 53% in TiO_2_/Cs NTs. However, in comparison to TiO_2_-NTs, drug release from CIP-loaded TiO_2_-NTs samples without a coating may last for 24 h at a rate of approximately 99.21%, whereas this rate is 66.19% in TiO_2_/Cs NTs.

### 3.6. Investigation of Chitosan-Coating Thickness on Release Behavior

**Figure 8:**
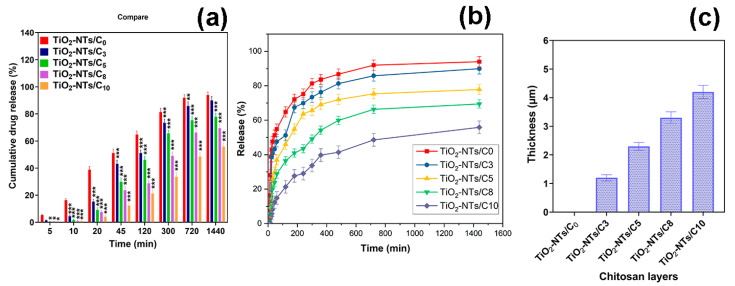
(**a**) Statistical effect of the thickness of the polymer on the release behavior of CIP. (**b**) Release profile of CIP from TiO_2_-NTs with different numbers of coating layers. (**c**) The coating thickness of the obtained samples with different polymer dipping processes (µm). (*** *p* < 0.001, ** *p* < 0.01, * *p* < 0.05).

Figure 8 compares the CIP drug release profiles of TiO_2_-NTs with and without Cs coating, and Table 4 lists the release percentages at various time intervals. The TiO_2-_NTs/C10, TiO_2_-NTs/C8, and TiO_2_-NTs/C5 samples showed burst release over the first hour and subsequent steady release, whereas the TiO_2_/C0-NTs and TiO_2_ NTs-C3-NTs samples released the drug almost immediately. Cs-coated TiO_2_-NTs had a significantly longer CIP release time than other samples. The Cs coatings became thicker as additional dipping cycles were applied, reducing the diffusion rate of CIP, and thus its release rate. These findings align well with prior studies, where the rate of drug elution was tuned by adjusting the thickness of polymer coating [23,33,34]. 

In addition, Table 4 also shows the encapsulation efficiency of CIP calculated from the releasing test. It was found that total drug loading into the samples decreased with an increase in the dipping/drying cycle [23].

### 3.7. Kinetic of Release 

As mentioned in Table 5, by employing kinetic equation models, it was determined that among different models, the drug release profile of the TiO_2_/Cs NTs fitted the Higuchi model very well, with a correlation coefficient (R^2^) value of 0.86.

### 3.8. Antibacterial Properties 

#### 3.8.1. Determination of Minimum Inhibitory Concentrations (MICs)

**Figure 9:**
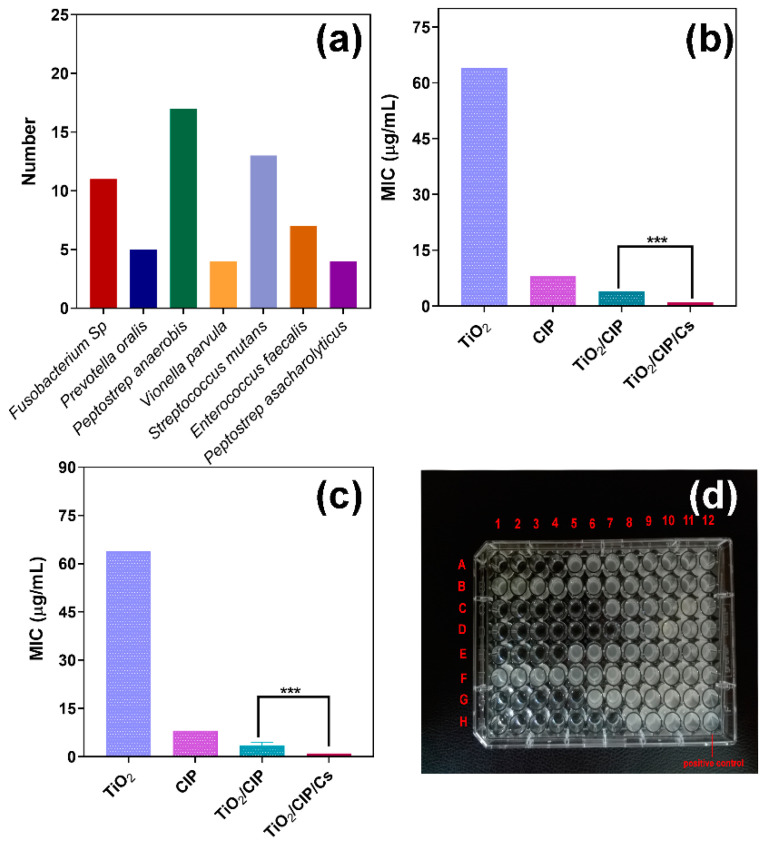
(**a**) Isolated bacteria; (**b**) MIC micro broth dilution of *Fusobacterium*; (**c**) MIC micro broth dilution of *Peptostreptococcus*; (**d**) the 96 well microplate broth microdilution method for the MIC assays with *Fusobacterium* and *Peptostreptococcus* strains. This plate has eight test rows (rows A, B, C, and D for *Peptostreptococcus* (from different sources); and rows E, F, G, and H for *Fusobacterium*) for MIC determinations of samples containing CIP at serial concentrations ranging from 0.06 to 64 µg/mL. Eight wells from each row in column 12 have been assigned as positive controls. (*** *p* < 0.001, ** *p* < 0.01, * *p* < 0.05).

Our research aimed to improve the antibacterial properties of TiO_2_-NTs, so TiO_2_-NTs, TiO_2_/CIP, and TiO_2_/CIP/Cs were immersed in the homogeneous bacteria liquid of *Peptostreptococcus* and *Fusobacterium* (10^6^ CFU/mL) to evaluate the antibacterial effects of the samples [23]. The MIC values of free CIP and TiO_2_/CIP/Cs against *Peptostreptococcus* and *Fusobacterium* were studied using a microtiter plate. CIP MIC analysis revealed that all *Peptostreptococcus* and *Fusobacterium* strains resulted in MIC values of 8 µg/mL (Table 6). However, 0.062–8 µg/mL of CIP did not have an inhibitory effect on bacterial growth. As a result, 8 µg/mL was chosen as the highest MIC value for CIP. Additionally, TiO_2_/CIP/Cs resulted in a 4 to 8-fold reduction in the MIC value when compared to free CIP, demonstrating that TiO_2_/CIP/Cs increased the antibacterial activity of CIP, considerably reducing the MIC values when compared to free CIP (Figure 9). Subsequently, the MICs of TiO_2_, TiO_2_/CIP, TiO_2_/CIP/Cs, and free CIP against *Peptostreptococcus* and *Fusobacterium* strains were determined. 

#### 3.8.2. Investigation of Minimum Bactericidal Concentrations (MBCs)

The antibacterial activities of all samples against *Fusobacterium* and *Peptostreptococcus* were determined using a broth microdilution assay (Table 7). After 48 h of incubation under aerobic conditions at 37 °C, colonies were noticed in the plates containing samples (TiO_2_, CIP, TiO_2_/CIP, TiO_2_/CIP/Cs), indicating the growth of bacteria. As presented in Table 7, colonies of *Fusobacterium* strain grew at the MIC concentration. For *Peptostreptococcus*, MICs of TiO_2_ and TiO_2_/CIP exhibited inhibition of bacterial growth. 

The MIC and MBC values of the TiO_2_-NTs were 64 μg/mL and those of the TiO_2_/CIP were 2  μg/mL against *Fusobacterium* and 4 μg/mL against *Peptostreptococcus*. Surprisingly, TiO_2_/CIP/Cs had a synergistic effect and had a MIC of 1  μg/mL. The bactericidal activity of the TiO_2_/CIP with MBCs of 2  μg/mL (*Fusobacterium*) and 1 μg/mL (*Peptostreptococcus*) was higher than that of CIP 8  μg/mL. These results thus confirm that the MBC against *Fusobacterium* and *Peptostreptococcus* is effective at dilutions of 8, 64, 2, and 1 (*Peptostreptococcus*) μg/mL; and 8, 64, 4, and 1 μg/mL (*Fusobacterium*) for CIP, TiO_2_-NTs, TiO_2_/CIP, and TiO_2_/CIP/Cs, respectively, which are consistent with the MIC results. The images of MBC plates are represented in Figures S1 and S2.

#### 3.8.3. Effect of TiO_2_-NTs on Biofilm Formation

**Figure 10:**
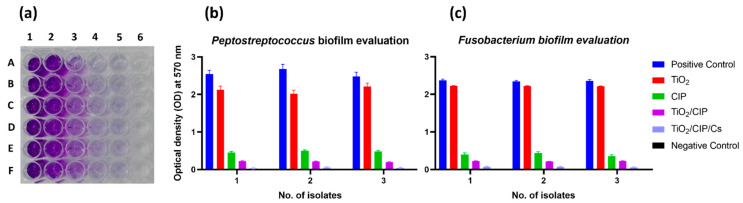
Detection of biofilm formation by *Fusobacterium* and *Peptostreptococcus* in the absence of samples. (**a**) A–C and D–F are related to isolates of *Peptostreptococcus* and *Fusobacterium*, respectively. Number 1 is a positive control, 2 is TiO_2_-NTs, 3 is CIP, 4 is TiO_2_/CIP, 5 is TiO_2_/CIP/Cs, and 6 is a negative control. (**b**,**c**) Antibiofilm activity of samples against *Peptostreptococcus* and *Fusobacterium,* respectively. The results are expressed as the means  ±  SD of triplicate assays from three independent experiments.

The inhibition of *Fusobacterium* and *Peptostreptococcus* biofilm formation by TiO_2_ and other nanocomposites was evaluated by crystal violet staining following growth in a 96-well microplate. The TiO_2_/CIP and TiO_2_/CIP/Cs were the most effective samples for preventing biofilm formation, whereas TiO_2_ was the least effective, showing no inhibition at the highest tested concentration (64 μg/mL) (Figure 10). Biofilm production strength was classified based on the optical density (OD) of the isolate: ≤0.232 for no biofilm detected, 0.232 < OD isolate ≤ 0.464 for weak biofilm formation, 0.464 < OD isolate ≤ 0.929 for moderate biofilm formation, and OD isolate > 0.929 for strong biofilm formation [50]. To compensate for background absorbance, the OD reading value (570 nm) of the blank was deducted from the sample values.

#### 3.8.4. Mechanism of Bactericidal Effect

TiO_2_-NTs can act as an effective drug reservoir to prevent bacterial colonization, implant infection, and cell adhesion [51]. The antimicrobial effect of TiO_2_/CIP/Cs is mostly attributable to CIP. CIP acts on bacterial topoisomerase II (DNA gyrase) and topoisomerase IV [52]. The targeting of CIP of the alpha subunits of DNA gyrase prevents it from supercoiling the bacterial DNA, inhibiting replication [53]. TiO_2_-NTs continuously release CIP in an aqueous microenvironment. However, Cs, which helps to maintain the release of CIP, also has an antibacterial effect [54]. The other reason for this antibacterial effect is the large surface area of TiO_2_-NTs, which leads to higher adhesion to bacteria cells [55,56]. 

The results demonstrate that bacterial cell adhesion (and hence biofilm formation) can effectively be controlled by tuning the wettability of TiO_2_-NTs via Cs coating [57]. 

The main reason for the bactericidal properties of TiO_2_/CIP/Cs is that it interferes with the integrity of the bacterial cell by binding to essential cellular structure [58]. TiO_2_-NTs also generate reactive oxygen species (ROS) and free radicals, which damage the bacterial cell wall and inhibit respiratory enzymes [59,60]. ROS affect bacterial cells by various mechanisms, leading to their death [61].

### 3.9. Hemocompatability Properties 

**Figure 11:**
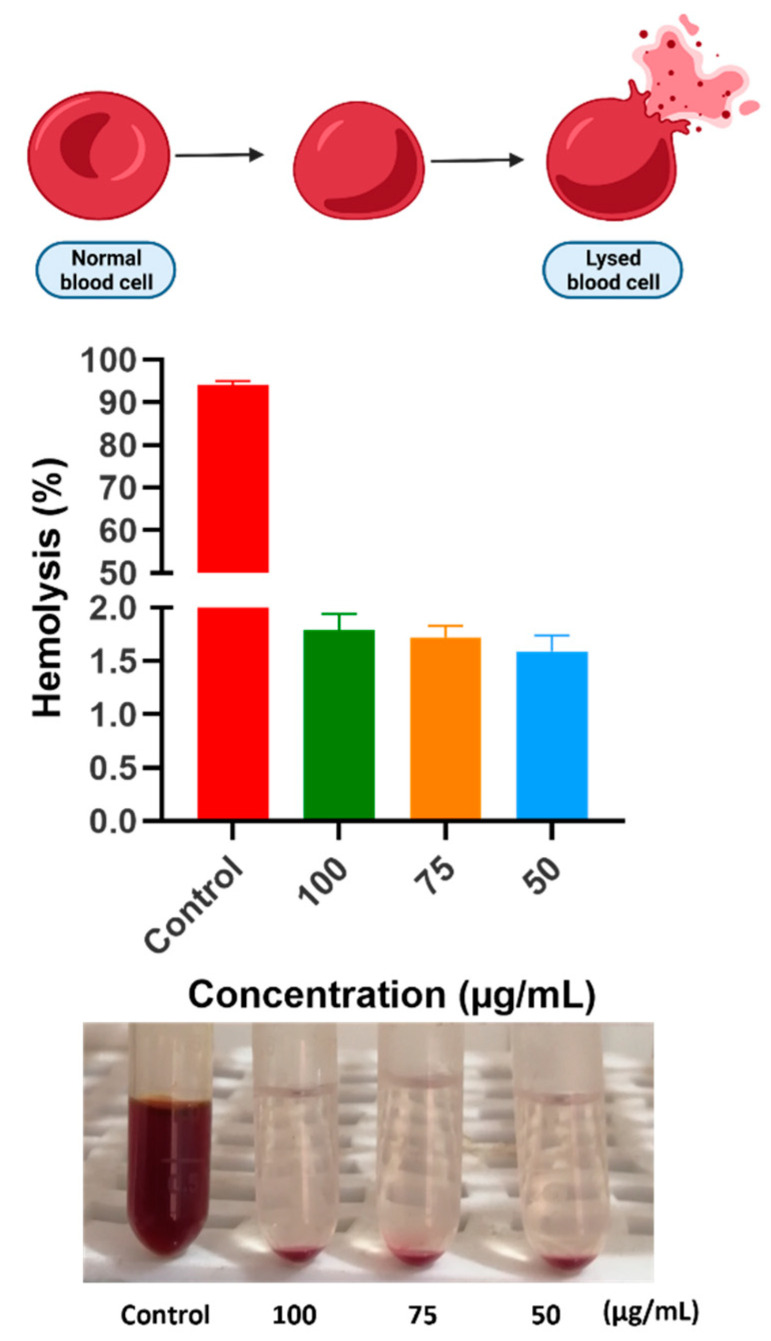
Hemolysis percentages of TiO_2_/CIP/Cs nanoformulations.

Materials intended for usage in the biomedical field must be examined carefully for their hemocompatibility. The morphology of RBCs or erythrocytes can be affected by nanoparticles, resulting in the lysis of RBCs, which is referred to as hemolysis. Figure 11 depicts the percentages of total hemolysis for TiO_2_/CIP/Cs. As seen in Figure 11, the hemoglobin in the control sample was released into the solution, turning the supernatant reddish after centrifugation [62]. According to hemolytic activity, all examined doses of TiO_2_/CIP/Cs samples can be categorized as safe and non-hemolytic (hemolytic rate below 2%) (100, 75, and 50 μg·mL^−1^) and thus can be used in the development of drug carriers.

## 4. Discussion

The results from characterization demonstrated that the TiO_2_-NTs samples containing CIP were successfully fabricated. Previous studies have proven that antiadhesive biomaterials are suitable to prevent peri-implant infection by inhibiting the production of bacterial biofilms from the beginning. This strategy has recently been explored, focusing on nanoscale manipulation of surface characteristics to selectively drive or hinder biofouling, which occurs prior to biofilm development [63]. This can be accomplished by modifying the physico-chemistry of a biomaterial’s surface layer via its wettability, chemical structure, topographical structure, and surface characteristics [64,65]. While it has been demonstrated that bacteria prefer hydrophobic surfaces, there is currently no clear pattern for their adhesion profile, which is particularly concerning given that intra-individual dental biofilms may contain over 700 species [66].

As mentioned in Table 3, a very high entrapment efficiency of drug, up to 93%, resulted from the smaller outer diameter and larger inner diameter of the nanotubes at T30%, which resulted in a higher drug capacity per unit weight than T15%–T25%. Due to its increased capacity, T30% may release a larger amount CIP than thinner TiO_2_-NTs/CIP [67]. In conclusion, larger nanotubes released a greater amount of drug. This is only partially due to increased entrapment efficiency. Indeed, between 1 and 2 h, the release from the larger nanotubes was significantly faster.

As presented in Figure 7, Cs coatings can provide the desired effect of sustained release. Likewise, TiO_2_-NTs loaded with gentamicin sulfate were covered with a thin layer film of Cs and gentamicin sulfate, which has high potential for maintaining drug release [22]. However, according to another study performed by Niu et al., it cannot improve as the coating becomes thicker. There were no noticeable differences between the data profiles for different coating layers in the subsequent release. Due to the hydrophilicity of Cs and the viscosity of its solution, the coating thickness increased noticeably during the dipping process at first, but then became unaffected as the dipping cycles multiplied. The drug release rate in this research was significantly faster than in previous investigations, and the total amount released over a 6-day period was greater than 90% in all groups [23].

The release mechanism was validated with the Higuchi model of release kinetics. This model is defined under sink conditions, which means that the release of the entirety of the drug in the solution does not exceed the saturation condition, leading to the precipitation of the drug. Numerous mathematical approaches were developed using Higuchi models to identify the possible drug release profiles of the active drugs included.

In the study conducted by Chennell et al., a comparable phenomenon was observed where the coating layer’s thickness and permeability to drug molecules were critical factors affecting drug release [68]. Figure 8 shows the effects of the chitosan coating on drug release. As shown, the desired sustained-release effect can be achieved. According to Feng et al., TiO_2_-NTs were coated with an efficient drug-release-sustaining thin film consisting of an outstanding blend of the two components, Cs and gentamicin sulfate. Increasing the thickness of the coating will not improve the situation. During a subsequent release, the data for TiO_2_-NTs/C0, TiO_2_-NTs/C3, TiO_2_-NTs/C5, TiO_2_-NTs/C8, and TiO_2_-NTs/C10 profiles did not differ significantly. Due to the hydrophilic nature of chitosan and the viscosity of its solution, the thickness of the coating initially increased, but this effect diminished as the dipping cycles increased in number [69]. Regarding determining a drug’s release, the thickness and permeability of the coating layer are critical [70,71]. The chemical composition, structure, and surface properties of the polymer play a role in this evaluation.

As shown by FTIR and EDX, CIP was successfully loaded into the TiO_2_-NTs. Cs, as a hydrophilic polymer, prevents and retards the passage of drug molecules from NTs to the solution. Niu et al. reported that due to low-molecular-weight Cs, the coating function is insignificant because of a shortage in chemical bonding between the molecules, resulting in better diffusion of PBS through the Cs layer [23]. In this case, in this study, high-molecular-weight Cs was used in five layers of coating. Regarding the antimicrobial experiment, there are two possible explanations for these findings. To begin, surface factors, such as structural, chemical, and physical properties, affect bacterial adhesion. Second, CIP is well known for its potent antibacterial properties [72,73].

As presented in Figure 9, both TiO_2_/CIP/Cs and TiO_2_/CIP have lower MIC values than free CIP. As a control, TiO_2_-NTs were used. It was determined that it had no antibacterial action against the strains of *Peptostreptococcus* and *Fusobacterium,* indicating that antibacterial activity resulted from CIP and Cs. Figure 8 reveals that TiO_2_/CIP coated with a polymer inhibited bacterial growth, particularly those adhering to the surface. These findings are in agreement with the results of the CFU count and the drug release assay. The increased antibacterial activity of TiO_2_-NTs loaded with antibiotics is consistent with prior studies. There are numerous processes by which polymers interact with bacterial cells, including contact release, fusion, and adsorption. When polymers come into contact with the wall of a bacterial cell, the drug is released into the bacterial cell, increasing the concentration of drug in the target region [74]. Simultaneously, the CFU counts in the TiO_2_/CIP and TiO_2_/CIP/Cs samples reduced considerably. Bacterial adhesion to implants was previously considered a necessary step in infection [69]. When the drug-coated TiO_2_-NTs samples were subjected to bacterial suspension, the CIP was released into the culture medium, physically interacting with the bacteria, and inhibited bacterial adherence. The CFU count decreased in the antibacterial environment.

This study was aimed at determining the MICs and MBCs of TiO_2_-NTs and its nanocomposites against *Fusobacterium* and *Peptostreptococcus*. This is the first study in the literature to include the MBCs of TiO_2_-NTs and its nanocomposites with Cs and CIP against *Fusobacterium* and *Peptostreptococcus*. The antibacterial effects of the samples were assessed by broth microdilution, MIC, and MBC tests. Serial dilutions of a solution are used for MIC to determine the lowest material concentration that would still show antibacterial properties. *Fusobacterium* and *Peptostreptococcus*, facultative anaerobic Gram-positive coccuses, were selected for use in this study because they are some of the facultative bacteria found in abscessed root canal cases and recurrent apical periodontitis [13], and can develop resistance to antimicrobial agents. 

Furthermore, exposure of a *Fusobacterium* biofilm to MBCs from all samples except TiO_2_ resulted in a decrease in biofilm viability. Similar observations have also been reported on biofilm formation and the killing of bacteria embedded in biofilms for *Peptostreptococcus*, another periodontopathogenic bacterial species. These results indicate that TiO_2_/CIP/Cs shows great promise as an antibiofilm agent.

## 5. Conclusions

The current study first synthesized TiO_2_-NTs via anodic oxidation. The roles of electrolyte percentage and viscosity in TiO_2_-NTs size was investigated. The synthesized NTs were found to be highly effective reservoirs for encapsulating CIP, a strong antibiotic, yielding high encapsulation efficiency of up to 93%. The NTs were coated with chitosan (up to 10 layers) to reduce the release rate and provide sustained release to reduce the side-effects associated with the systemic administration of CIP. It was found that the higher the number of Cs coats, the lower the rate of release (i.e., more sustained release). The prepared formulation exhibited a synergistic antibacterial effect on Gram-positive and negative bacteria. Since pure NTs (unloaded with CIP and uncoated with Cs) had no antibacterial activity, the synergistic effect was attributed to the simultaneous presence of both Cs and CIP. Overall, the current study presents a simple, cost-effective approach for developing biocompatible implants with synergistic antibacterial effects. Future studies could focus on bone integration of the formulation and its compatibility with bone tissues, which could be achieved through further surface modification techniques. Additionally, fixation of Cs chains through chemical (e.g., glutaraldehyde) or physical (e.g., phosphate-based linkers) crosslinking could further extend the release period of CIS from the formulation. Furthermore, the antibiotics can be grafted/conjugated onto the polymer backbone to enhance its antibacterial properties through chemical reactions. Ultimately, the development of such formulations is aimed at enhancing the therapeutic efficacy of antibiotics for dental implants, which could overcome the antibiotic resistance while decreasing the antibiotic dose through such synergistic effects and sustained release.

## Figures and Tables

**Table 1:** Energy dispersive X-ray spectroscopy (EDX) analysis of all samples.

Sample	Element	Mass Percent (%)	Atom Percent (%)	abs. Error [%] (1 Sigma)
**TiO_2_-NTs**	O	41.34	64.29	8.82
	Ti	27.94	14.52	0.85
	Ca	23.20	14.40	0.72
	Na	3.96	4.28	0.38
	Cl	3.57	2.50	0.19
**TiO_2_/CIP**	O	44.39	58.30	9.23
	C	10.36	18.12	2.93
	Ti	22.88	10.04	0.82
	Ca	16.20	8.49	0.62
	Na	3.99	3.65	0.39
	Cl	2.01	1.19	0.13
	F	0.19	0.21	0.31
**TiO_2_/CIP/Cs**	C	40.71	51.99	10.03
	O	33.82	32.43	9.21
	N	5.05	5.53	3.59
	F	4.91	3.97	2.39
	Ca	5.37	2.06	0.31
	Cl	3.92	1.70	0.25
	Ti	5.22	1.67	0.32
	Na	1.00	0.67	0.16

**Table 2:** The effect of increasing the water percentage in the electrolyte on the structure of TiO_2_-NTs at 50 V, with 0.35 wt.% ammonium fluoride and ethylene glycol for 2 h at room temperature.

Water Content (*w*/*w*%)	15	20	25	30
Code	T15%	T20%	T25%	T30%
Nanotube wall thickness (nm)	5.92	8.77	9.53	15.8
Inner diameter of nanotubes (nm)	59.92	72.64	66.3	84.61
Nanotube length (µm)	1.56	2.01	2.08	3.21

**Table 3:** Entrapment efficiency of CIP-loaded TiO_2_-NTs prepared at 50 V in an electrolyte containing 15, 20, 25, or 30% water; 0.35% ammonium; and ethylene glycol for 2h.

Sample	Entrapment Efficiency (EE)%
T15% (*w*/*w*)	83.19 ± 1.68
T20% (*w*/*w*)	83.70 ± 1.77
T25% (*w*/*w*)	86.48 ± 1.4
T30% (*w*/*w*)	92.95 ± 2.25

**Table 4:** The thickness of Cs coating and cumulative release data of CIP from TiO_2_-NTs. Abbreviations: SD = standard deviation, EE = encapsulation efficiency.

Samples	Chitosan Layers	Thickness (µm)		Release Profile (%)								EE (%)
				5 min	10 min	20 min	45 min	120 min	300 min	720 min	1440 min	
TiO_2_ NTs/C0	0	mean	0	5.35	16.38	38.8	51.23	64.76	81.32	91.94	93.96	83.19
		SD	0	0.65	1.21	2.34	2.79	2.66	2.85	2.31	2.35	1.12
TiO_2_-NTs/C3	3	mean	1.2	1.42	4.41	15.32	43.17	51.23	73.4	85.83	89.9	87.68
		SD	0.12	0.35	0.92	1.58	2.65	2.15	2.75	3.05	2.93	1.85
TiO_2_-NTs/C5	5	mean	2.3	0.4	1.63	9.15	29.82	46.02	65.68	75.32	77.8	90.22
		SD	0.23	0.22	0.68	1.59	2.99	2.64	2.21	2.8	2.56	2.59
TiO_2_-NTs/C8	8	mean	3.3	0.47	0.95	7.52	23.74	28.77	49.05	66.31	69.45	92.04
		SD	0.16	0.35	0.58	1.25	2.89	2.6	2.85	2.75	2.64	1.48
TiO_2_-NTs /C10	10	mean	4.2	0.18	0.87	4.1	12.36	21.29	33.67	48.6	55.83	93.97
		SD	0.24	0.54	0.3	1.05	2.1	2.54	2.94	2.73	2.37	1.21

**Table 5:** The models of kinetic release and the parameters determined for TiO_2_/CIP and TiO_2_/CIP/Cs formulation (TiO_2_-NTs/C10).

Release Model	Equation	R^2^	
		without Chitosan (A)	with Chitosan (B)
Zero-order	C_t_ = C_0_ + K_0_t	R^2^ = 0.4159	R^2^ = 0.5444
Korsmeyer-Peppas	M_t_/M_∞_ = K_t_^n^	R^2^ = 0.5542 N = 0.3953	R^2^ = 0.6811 N = 0.5234
First-order	LogC = LogC_0_ + K_t_/2.303	R^2^ = 0.8667	R^2^ = 0.6565
Higuchi	$Q=K_{H}\surd t$	R^2^ = 0.6821	R^2^ = 0.8018

**Table 6:** MIC values of samples against *Peptostreptococcus* and *Fusobacterium* bacteria.

Sources.	Strain	CIP (µg/mL)	TiO_2_ (µg/mL)	TiO_2_/CIP (µg/mL)	TiO_2_/CIP/Cs (µg/mL)
1	*Peptostreptococcus*	8 ± 0	64 ± 0	2 ± 0	1 ± 0
2	*Peptostreptococcus*	8 ± 0	64 ± 0	2 ± 0	1 ± 0
3	*Peptostreptococcus*	8 ± 0	64 ± 0	2 ± 0	1 ± 0
4	*Peptostreptococcus*	8 ± 0	64 ± 0	2 ± 0	1 ± 0
5	*Peptostreptococcus*	8 ± 0	64 ± 0	2 ± 0	1 ± 0
6	*Peptostreptococcus*	8 ± 0	64 ± 0	2 ± 0	1 ± 0
1	*Fusobacterium*	8 ± 0	64 ± 0	4 ± 0	1 ± 0
2	*Fusobacterium*	8 ± 0	64 ± 0	4 ± 0	1 ± 0
3	*Fusobacterium*	8 ± 0	64 ± 0	4 ± 0	1 ± 0

**Table 7:** MIC and MBC (μg/mL) values of TiO_2_, CIP, TiO_2_/CIP, and TiO_2_/CIP/Cs against *Fusobacterium* and *Peptostreptococcus*.

Bacterial Strain	Sample	MIC (μg/mL)	MBC (Pour Plate) (μg/mL)
***Fusobacterium***	TiO_2_-NTs	64	64
	CIP	8	16
	TiO_2_/CIP	2	2
	TiO_2_/CIP/Cs	1	2
***Pepto streptococcus***	TiO_2_-NTs	64	64
	CIP	8	8
	TiO_2_/CIP	4	4
	TiO_2_/CIP/Cs	1	1

## Data Availability

The data presented in this study are available on request from the corresponding author.

## Supplementary Materials

The following supporting information can be downloaded at: https://www.mdpi.com/article/10.3390/pharmaceutics14071359/s1, Figure S1: Evaluation of MBC values of tested (a) CIP, (b) TiO_2_ NTs, (c) TiO_2_/CIP, and (d) TiO_2_ NTs/CIP/Cs in diferent concentrations against *Peptostreptococcus,* Figure S2: Evaluation of MBC values of tested (a) CIP, (b) TiO_2_ NTs, (c) TiO_2_/CIP, and (d) TiO_2_ NTs/CIP/Cs in diferent concentrations against *Fusobacterium*.
