# Supplementary material for: Ciprofloxacin-Loaded Titanium Nanotubes Coated with Chitosan: A Promising Formulation with Sustained Release and Enhanced Antibacterial Properties
Source: Pharmaceutics. 2022 Jun 27;14(7):1359. doi: 10.3390/pharmaceutics14071359 (PMC9316085; doi:10.3390/pharmaceutics14071359)
Supplement: Supplementary file 1 [file pharmaceutics-14-01359-s001.zip › pharmaceutics-1740356-supplementary.pdf]

pharmaceutics

MDPI

Supporting information

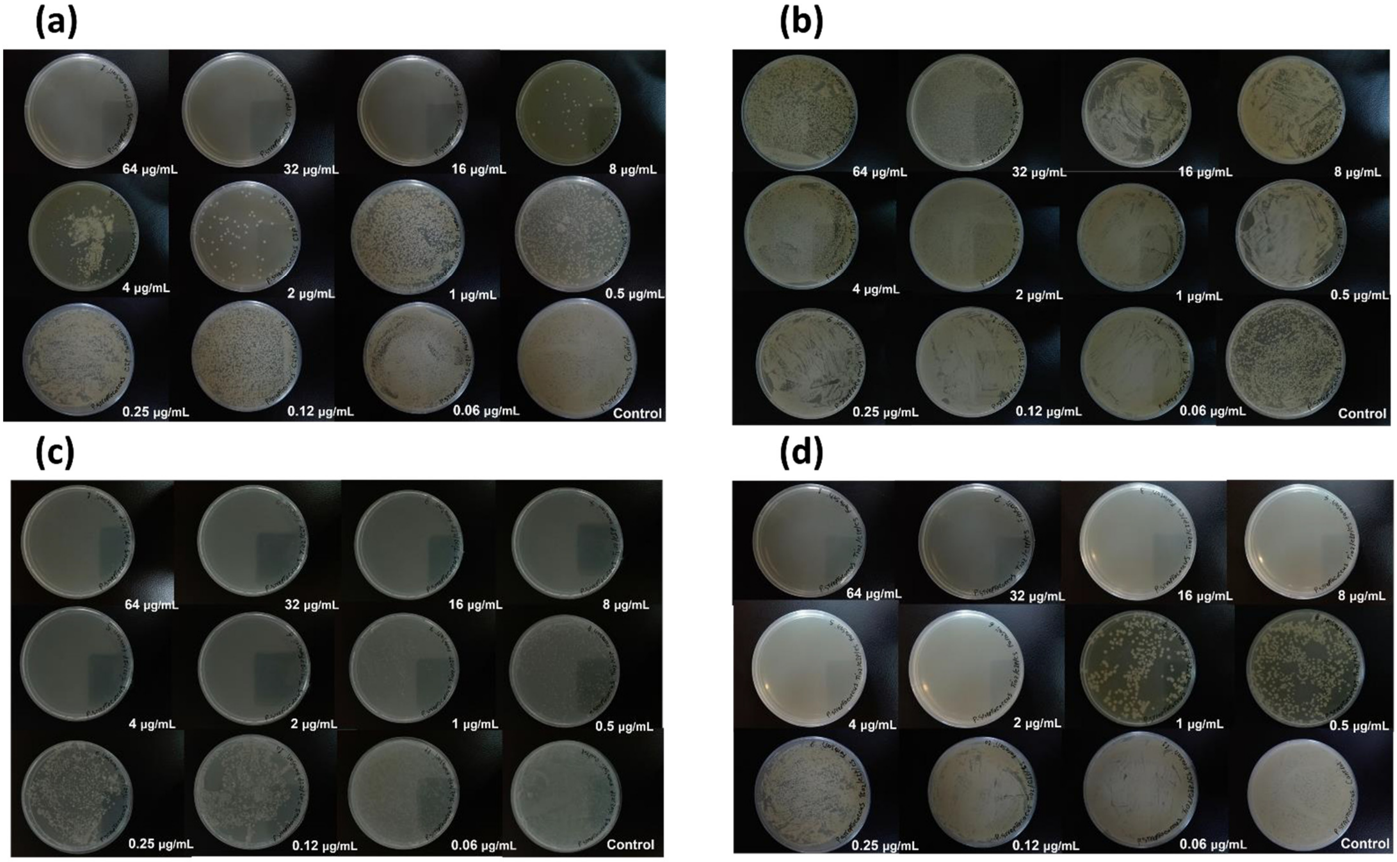

Figure S1. Evaluation of MBC values of tested a) CIP, b) TiO_2 NTs, c) TiO2/CIP, and d) TiO2 NTs/CIP/Cs in diferent concentrations against *Peptostreptococcus*

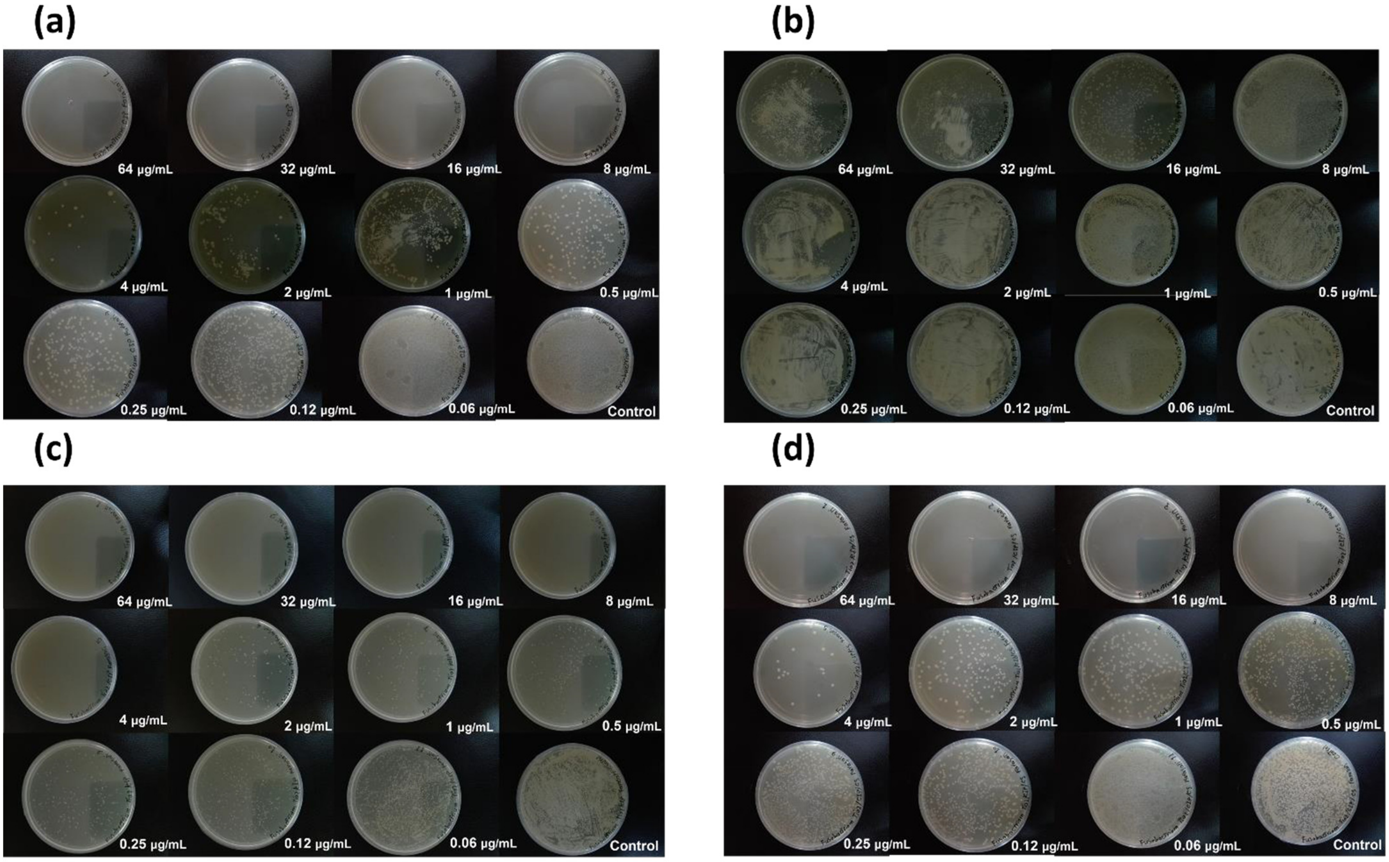

Figure S2. Evaluation of MBC values of tested a) CIP, b) TiO_2 NTs, c) TiO2/CIP, and d) TiO2 NTs/CIP/Cs in diferent concentrations against *Fusobacterium.*
